# Anemia in Pregnancy With CKD

**DOI:** 10.1016/j.ekir.2024.01.015

**Published:** 2024-01-11

**Authors:** Margriet F.C. de Jong, Elizabeta Nemeth, Pien Rawee, Kate Bramham, Michele F. Eisenga

**Affiliations:** 1 Department of Internal Medicine, Division of Nephrology, University Medical Center Groningen, The Netherlands; 2 Department of Medicine, University of California, Los Angeles, California, USA; 3 Department of Women and Children’s Health, King’s College London, London, UK

**Keywords:** anemia, CKD, hepcidin, iron, kidney disease, pregnancy

## Abstract

Chronic kidney disease (CKD), anemia, and iron deficiency are global health issues affecting individuals in both high-income and low-income countries. In pregnancy, both CKD and iron deficiency anemia increase the risk of adverse maternal and neonatal outcomes, including increased maternal morbidity and mortality, stillbirth, perinatal death, preterm birth, and low birthweight. However, it is unknown to which extent iron deficiency anemia contributes to adverse outcomes in CKD pregnancy. Furthermore, little is known regarding the prevalence, pathophysiology, and treatment of iron deficiency and anemia in pregnant women with CKD. Therefore, there are many unanswered questions regarding optimal management with oral or i.v. iron and recombinant human erythropoietin (rhEPO) in these women. In this review, we present a short overview of the (patho)physiology of anemia in healthy pregnancy and in people living with CKD. We present an evaluation of the literature on iron deficiency, anemia, and nutritional deficits in pregnant women with CKD; and we evaluate current knowledge gaps. Finally, we propose research priorities regarding anemia in pregnant women with CKD.

See Commentary on Page 1152

CKD, anemia, and iron deficiency are global health issues affecting individuals in both high-income and low-income countries, amounting to >800 million individuals with CKD and approximately 1.2 billion individuals with iron deficiency anemia.^1^^,^^2^ CKD is also associated with iron deficiency and anemia. Prevalence of iron deficiency and anemia in CKD may reach 73% and 63%, respectively, depending on CKD stage.^3^, ^4^, ^5^ A unique subgroup among patients with CKD is the population of pregnant women with CKD, especially when considering anemia and iron deficiency. The prevalence of CKD in women of reproductive age has previously been shown to be 0.5% to 6% in high-income countries and up to 9% in low-income countries.^6^, ^7^, ^8^, ^9^ Pregnancy in women with CKD, including kidney transplant recipients, is likely to be less common^9^; however, similar to pregnancies with iron deficiency anemia, it is associated with increased risk of adverse maternal and fetal outcomes, such as hypertensive disorders of pregnancy, including preeclampsia, preterm birth, low birthweight, admission to the neonatal intensive care unit, impaired immune function, and neurodevelopmental impairment, which may possibly also affect the offspring later in life.^1^^,^^10^, ^11^, ^12^, ^13^, ^14^, ^15^, ^16^, ^17^, ^18^, ^19^, ^20^, ^21^, ^22^, ^23^, ^24^, ^25^, ^26^, ^27^ Moreover, during pregnancy in general, the prevalence of iron deficiency anemia is particularly high because of the increased maternal, placental, and fetal demand for iron.^1^^,^^23^^,^^28^, ^29^, ^30^, ^31^, ^32^

However, to date, little is known regarding the prevalence, pathophysiology, and treatment of iron deficiency and anemia in pregnant women with CKD.^33^^,^^34^ Therefore, many questions have not been answered for pregnant women with anemia and CKD, including the underlying pathophysiology, target levels of hemoglobin (Hb) and iron parameters, when treatment is needed, and what optimal dosing schedules of oral or i.v. iron and rhEPO are. In the current review, we present a short overview of the (patho)physiology and treatment of anemia in healthy pregnancy and in CKD condition, and review the current knowledge related to pregnancy with CKD. We describe evidence gaps and hypotheses following from the (patho)physiology of healthy pregnancy and CKD. Finally, we propose research priorities regarding anemia in pregnant women with CKD.

### Anemia in Healthy Pregnancy

#### Erythropoiesis and Iron Homeostasis in Pregnancy

During healthy pregnancy, erythropoiesis and iron homeostasis undergo a profound change (Figure 1). A physiological decrease in Hb concentration occurs due to hemodilution and an assumed decrease in erythrocyte life span, partially offset by an increase in erythropoietin (EPO) concentrations and erythrocyte mass.^35^, ^38^, ^39^, ^40^ Furthermore, iron deficiency is an important contributing factor to the anemia of pregnancy, because iron utilization for maternal red blood cell expansion and fetoplacental growth exceeds the supply of iron from diet (Table 1).^32^^,^^41^, ^42^, ^43^ The iron requirements gradually increase during gestation, from an additional daily need of 0.8 mg of iron in the first trimester to >6 mg in the third trimester, attributed equally to increased demands of mother and fetus.^29^^,^^32^ During pregnancy, iron is efficiently mobilized from maternal stores, as reflected by the gradual decrease in serum ferritin over the course of the pregnancy (Figure 1); however, many women enter pregnancy with minimal iron stores. Recycling of maternal red blood cell iron serves as a substantial source of iron for the fetus, particularly in women with depleted body iron stores.^44^ Soluble transferrin receptors (sTfRs) levels, reflecting the mass of erythroid precursors and their iron status, are increased in the third trimester of pregnancy, particularly in women with iron deficiency anemia.^45^

**Figure 1:**
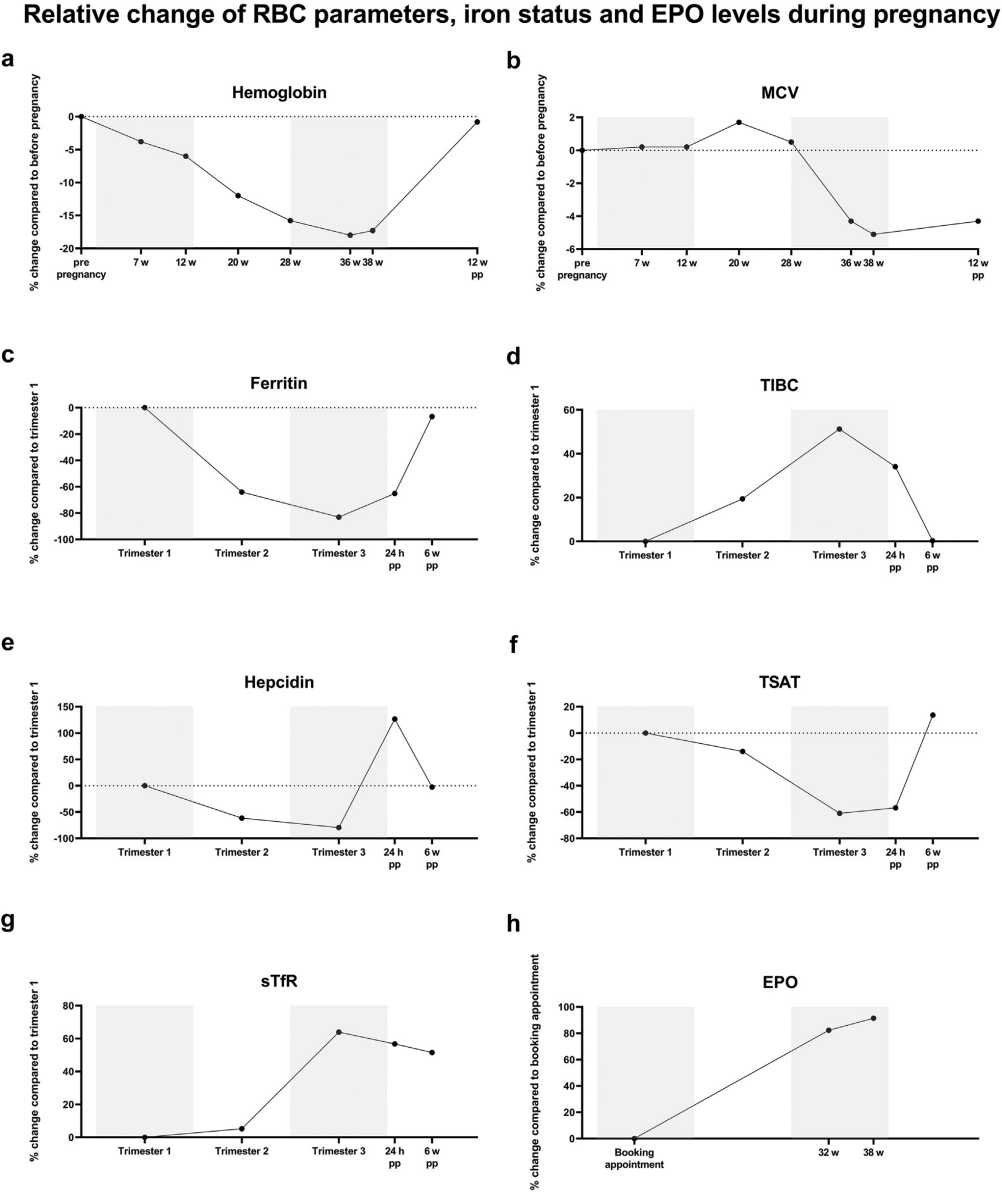
Course of iron and red blood cell parameters during healthy pregnancy. Reproduced from reference McMullin *et al.*,^35^ van Santen *et al.*,^36^ and Whittaker *et al.*^37^ with permission.

**Table 1:** Iron balance in pregnancy

Iron fate	Amount, mg
Fetal iron	270
Placental iron	90
Baseline maternal body iron loss	230
Expansion of maternal RBC mass	450
Total iron needs during pregnancy	1040
RBC-mass contraction after delivery (450 mg) minus the	
blood lost at delivery (150 mg)	−300
Net pregnancy iron loss to the mother	740

RBC, red blood cell.

All values are means. Adapted from reference Fisher *et al.*^30^ with permission.

At present, we know that coordination between erythropoietic activity and iron homeostasis is mainly regulated by 3 hormones, namely EPO, hepcidin, and erythroferrone (ERFE).^3^^,^^30^^,^^46^^,^^47^ Major alterations occur in these 3 hormones during pregnancy (Figure 2). First, peak EPO concentration is reached during the third trimester but continues to be elevated postpartum.^48^ Second, maternal hepcidin is known to be decreased in the second and third trimester of the pregnancy by a yet unknown mechanism, although iron deficiency certainly contributes. Hepcidin coordinates iron homeostasis by directly binding to ferroportin, the sole known iron exporter, leading to blocking of iron absorption from the gut and iron release from stores in the reticuloendothelial system.^3^^,^^49^ As such, lowering of maternal hepcidin concentrations during pregnancy creates a milieu of higher iron availability to the placenta and the fetus.^30^^,^^36^, ^50^, ^51^, ^52^, ^53^ Mouse models show that low maternal hepcidin levels are critical for establishing normal fetal iron endowment.^51^ Third, ERFE levels mildly increase during pregnancy and are positively associated with EPO and sTfR levels. ERFE is produced by erythroblasts in response to EPO. ERFE decreases hepatic expression of hepcidin and thereby increases circulating iron concentrations.^46^^,^^54^ However, in animal studies of nonanemic pregnancies, ERFE did not appear to play a major role in maternal and fetal iron homeostasis.^55^ Finally, in case of maternal iron deficiency anemia, the common homeostatic regulatory mechanisms of iron uptake and transfer to the fetus are engaged and possibly regulated more by fetal than by maternal iron demands.^56^ Maternal and fetal hepcidin both decrease further with iron deficiency anemia to promote iron availability and transfer to the fetus. Interestingly, placenta senses its own iron levels via the iron-regulatory protein 1 and, in conditions of severe iron deficiency, modulates its iron transporters to acquire and retain iron, maintaining its own iron homeostasis.^57^^,^^58^

**Figure 2:**
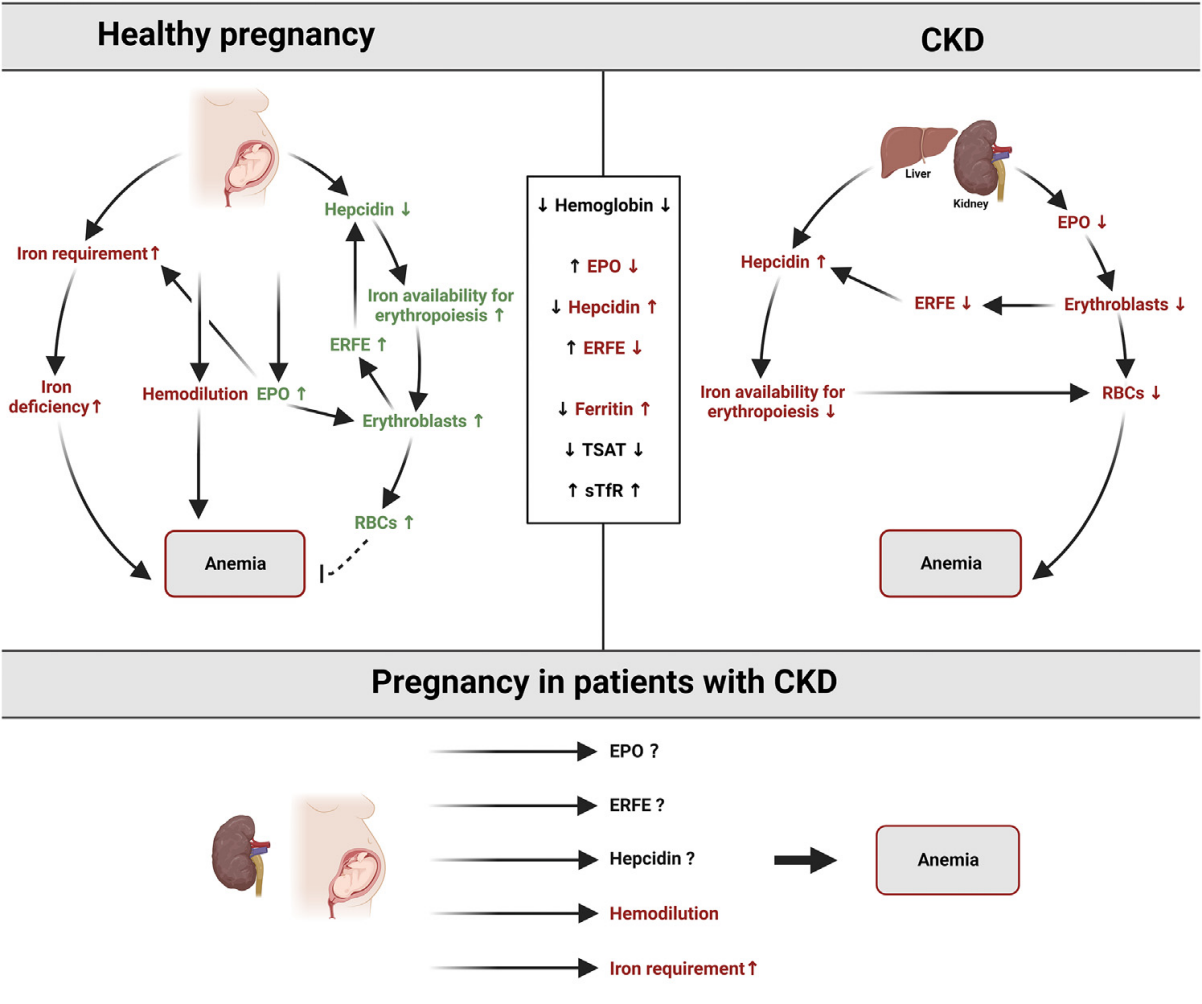
Simplified pathophysiology of iron deficiency anemia in healthy pregnancy, in CKD, and in pregnant women with CKD. CKD, chronic kidney disease; EPO, erythropoietin; ERFE, erythroferrone; IDA, iron deficiency anemia; RBC, red blood cell. Red color: process that worsens anemia; green color: process that diminishes anemia. Created with Biorender.com.

#### Definitions and Treatment of Anemia and Iron Deficiency During Pregnancy

To define anemia in pregnancy, World Health Organization, American College of Obstetricians and Gynecologists and the British Society for Haematology practice guidelines agreed on existing thresholds in the pregnant population (Table 2); however, more evidence is needed to link Hb levels with specific clinical outcomes in pregnancy.^59^, ^60^, ^65^, ^66^, ^67^, ^68^ This is further confounded by a considerable interpersonal variation in the degree of plasma volume expansion.^31^^,^^59^, ^60^, ^65^, ^66^^,^^69^ In fact, the lack of anemia caused by the absence of hemodilution has been found to be prognosticator of worse outcomes,^65^ implying that physiological anemia due to hemodilution should be regarded as beneficial.^70^^,^^71^ However, anemia due to other causes, e.g., iron deficiency, is considered detrimental and warrants treatment. Definition of iron deficiency in healthy pregnancy is based on the general population cutoffs and is usually defined as ferritin levels <30 μg/l and low transferrin saturation (TSAT) <20%; however, research on pregnancy-specific thresholds of serum ferritin concentrations across gestation is lacking.^59^, ^60^, ^61^ Anemia and iron deficiency during pregnancy are generally managed by oral or i.v. iron. It has been shown that daily versus intermittent iron supplementation leads to similar maternal and infant outcomes at birth; however, alternate-day supplementation was associated with fewer side effects and increased iron bioavailability than twice-daily or consecutive-day dosing.^72^, ^73^, ^74^ I.V. iron should be used from the second trimester onward up to postpartum, in case of intolerance or nonresponsiveness to oral iron, or when there is a clinical need for rapid and efficient anemia treatment.^75^^,^^76^ Treatment with rhEPO is mainly used when anemic women during or after pregnancy decline blood transfusion.^77^

**Table 2:** Definitions of anemia and iron target levels according to guidelines

	Pregnancy		Pregnancy in CKD			
	US guidelines^59^	UK guidelines^60^	Dutch guidelines^61^	UK guidelines^62^	Italian guidelines^63^	German guidelines^64^
T1 Hb	<11 g/dl	<11 g/dl	<11 g/dl	10.5–11.0 g/dl	Stage 5D: 10–11 g/dl	<10–10.5 g/dl
				depending on gestation	all trimesters	all trimesters
T2 Hb	<10.5 g/dl	<10.5 g/dl	<10.0 g/dl			
T3 Hb	<11 g/dl	<10.5 g/dl	<10.0 g/dl			
Postpartum Hb		<10.0 g/dl				
Ferritin	≤30 μg/l	<30 μg/l	stage 1–2: ≤80 μg g/l	<100 μg/l		
			stage 3–5 ND: ≤200 μg g/l			
TSAT	<16%		Stage 3–5 ND: ≤30%	<20%	stage 5 D: ≤30%	
HRC				>6%		
CHr				<25 pg		

CHr**,** reticulocyte hemoglobin content; D, dialysis; Hb, hemoglobin; HRC, hypochromic red blood cells; ND, nondialysis; T, trimester; TSAT, transferrin saturation; UK, United Kingdom; US, United States of America; WHO, World Health Organization.

### Anemia in CKD

Anemia in CKD has a multifactorial cause, including a relative EPO deficiency, iron deficiency, uremic-induced inhibitors of erythropoiesis, and shortened red blood cell lifespan.^3^ Iron deficiency can be absolute or functional. Absolute iron deficiency implies a deficit of total body iron, whereas functional implies a deficiency of circulating iron that limits erythropoiesis despite normal or elevated body iron stores.^3^ The latter is mainly caused by increased levels of hepcidin, inflammatory cytokines, and, if applicable, use of erythropoiesis stimulating agents.^3^^,^^78^ Hepcidin excess, often present in CKD due to the reduced renal clearance and up-regulation of hepcidin by proinflammatory cytokines, leads to impaired iron absorption from the gut and reduced iron mobilization from stores. Anemia and iron deficiency in CKD are generally defined according to the definitions used in Table 3, although these cutoffs have come under scrutiny and discussion. In addition, the current definitions for iron deficiency are mainly based on ferritin and TSAT levels; however, other iron status parameters such as sTfR levels, percentage hypochromic red blood cells, or reticulocyte Hb content may help define the conditions better or evaluate the efficacy of iron therapy, although they are mostly used in the academic setting.^81^, ^82^, ^83^ Controversy also exists regarding how to manage iron metabolism in CKD and therefore management and guidelines among countries have differences (Table 4).^78^ Data on clinical benefits of iron administration in CKD beyond stimulating erythropoiesis are limited and rarely assessing hard patient outcomes.^3^^,^^78^^,^^84^ Nevertheless, i.v. iron formulations have been found to be superior to oral iron in raising Hb levels, and delaying or reducing the need for other anemia management, including need for erythropoiesis stimulating agent therapy.^85^^,^^86^ Regarding these outcomes, the FIND-CKD study has established in the nondialysis CKD population that i.v. iron (ferric carboxymaltose) dosed to a target ferritin of 400 to 600 μg/l was superior to i.v. iron dosed to a target ferritin of 100 to 200 μg/l or oral iron.^84^ In a dialysis-dependent CKD population, the PIVOTAL study showed that proactive i.v. iron (iron sucrose) administration (unless serum ferritin >700 μg/l or TSAT >40%) was superior to a reactive strategy (triggered only for TSAT <20% and ferritin <200 μg/l) with respect to a lower risk of death or major cardiovascular event, lower dose of erythropoiesis stimulating agents needed, and a lower incidence of blood transfusions; whereas no difference was found for incidence of infections or hospitalizations.^87^ Finally, in patients with CKD, blood transfusions are generally avoided to minimize the general risks related to its use, especially in patients eligible for organ transplantation due to induction of HLA antibody.^88^

**Table 3:** Definitions used for anemia and iron deficiency in CKD

	WHO^1^	KDIGO^3^	ERBP^79^	NICE^80^
Anemia	M: Hb <13 g/dl			
	F: Hb <12 g/dl			
Absolute ID				
-(ND-CKD)		TSAT <20% + Fe <100 μg/l		
-(DD-CKD)		TSAT <20% + Fe <200 μg/l		
Functional ID				
-(ND-CKD)		TSAT <20% + Fe >100 μg/l		
-(DD-CKD)		TSAT <20% + Fe >200 μg/l		
Iron deficiency				
-if ESA naive			TSAT <20% + Fe <100 μg/l	
-if on ESAs			TSAT ≤30% +Fe ≤300 μg/l	
-in general				Use %HRC >6%, only if blood processing within 6 hours; if not possible, use CHr <29 pg; If not, TSAT <20% with Fe <100 μg/l

CHr, reticulocyte hemoglobin content; CKD, chronic kidney disease; DD-CKD: dialysis-dependent chronic kidney disease; ERBP, European Renal Best Practices; ESA, erythropoietin stimulating agent; F, female; Fe, ferritin; Hb, hemoglobin; HRC, hypochromic red blood cells; ID, iron deficiency; KDIGO, Kidney Disease Improving Global Outcomes; M, male; ND-CKD, nondialysis chronic kidney disease; NICE, National Institute for Health and Care Excellence; T, trimester; TSAT, transferrin saturation; UK, United Kingdom; US, United States of America; WHO, World Health Organization.

**Table 4:** Treatment targets of anemia in CKD according to the different current CKD guidelines

	KDIGO^3^	ERBP^79^	NICE^80^
Hb target during ESA treatment	<11.5 g/dl >11.5 g/dl for more QoL. Avoid >13 g/dl	10–12 g/dl	10–12 g/dl
Start of iron therapy	If Hb increase/ESA dose reduction is desired and ferritin ≤500 μg/l and TSAT ≤30%	When ID is present as defined in Table 3	Ferritin <100 g/dl and TSAT <20%
Ferritin and TSAT targets	Does not recommend routine use of iron if ferritin >500 μg/l or TSAT >30%	Avoid SF >500 ng/ml and TSAT >30%.	Avoid SF >800 μg/l Review iron dose when ferritin >500 μg/l

CKD, chronic kidney disease; ERBP, European Renal Best Practice; ESA, erythropoietin stimulating agent; Hb, hemoglobin; ID, iron deficiency; KDIGO, Kidney Disease Improving Global Outcomes; NICE, National Institute for Health and Care Excellence; QoL, quality of life; SF, serum ferritin; TSAT, transferrin saturation.

### Anemia in Pregnancy and CKD

Anemia is common in pregnancy and in CKD and, therefore, likely to be prevalent when the 2 conditions coexist.

#### Definitions of Anemia and Iron Deficiency in Pregnant Women With CKD

Definitions of anemia and iron deficiency for the specific population of pregnant women with CKD are lacking. Definitions for healthy pregnancy or nonpregnant CKD might not be valid because pregnancy in CKD is probably characterized by a different pathophysiology (see also next paragraph). Indeed, to date, no studies have been performed to identify optimal target values of Hb and iron parameters in pregnant women with CKD relating to outcomes,^33^ and these are likely to be challenging due to population heterogeneity and prescribing practice. However, guidelines on pregnancy and CKD give advice about target levels for Hb and iron parameters although they report that sensitivity and specificity of these parameters are unknown in this patient setting (Table 2).^61^^,^^62^ Similar to the definitions of iron deficiency in healthy pregnancy, which are based on the general population cutoffs,^59^^,^^60^ current advice for definitions for pregnant women with CKD are derived from those in the general pregnant population and the CKD population (Tables 2 and 3).^61^, ^62^, ^63^^,^^89^ For Hb and hematocrit, target levels 10.0 to 11.0 g/dl and 30% to 35% have been recommended for pregnant women with CKD with or without dialysis.^60^^,^^63^, ^89^, ^90^, ^91^, ^92^ Regarding definitions for different CKD stages, the Dutch guideline is the only guideline presenting advice on different definitions across CKD stages 1 to 2, 3 to 5 nondialysis, and dialysis. It is important to note that there is no evidence-based rationale for these treatment target levels because of a lack of research on cutoffs relating to outcomes. For example, in the Dutch guideline on CKD and Pregnancy the target values were based on frequency of anemia in the specific CKD stages compared with the general population.^61^ To further complicate the field of definitions derived from the general and CKD populations, it can be argued that definitions in pregnant women with CKD should not only differ among CKD stages, but also among trimesters of pregnancy. For example, in healthy pregnancy, high ferritin levels in the second or third trimester are known to be associated with poorer pregnancy outcomes, including preterm birth and low birthweight.^93^, ^94^, ^95^

#### Pathophysiology of Anemia in Pregnant Women With CKD

In Figure 2, we summarize the presumed pathophysiology of anemia in pregnant women with CKD. The pathophysiology of anemia in this specific population of pregnant women with CKD has not been investigated, but it is multifactorial and can at least partly be described as extrapolated from the pathophysiology of anemia in healthy pregnancy and in CKD. For example, apart from representing high iron stores, ferritin is an acute-phase reactant whose levels increase in response to inflammation.^96^^,^^97^ Adjustment of serum ferritin for inflammation has not been validated in pregnant women.^97^^,^^98^ Moreover, pregnancy itself might be associated with a physiological rise in acute-phase proteins (C-reactive protein, alfa-1-antichymotrypsin) and increased iron utilization, both of which influence serum ferritin levels in an opposing manner.^99^ Finally, iron deficiency during pregnancy could also be identified by increased sTfR concentrations; however, these are not routinely assessed in clinical settings yet. Notably, neither trajectories of routinely tested variables (Hb, ferritin, TSAT, iron concentrations, and inflammatory parameters) nor variables used in research (hepcidin and sTfR) have been investigated in pregnant patients with CKD. Therefore, in Figure 2, we show which regulatory mechanisms are opposite in the pathophysiology of anemia in healthy pregnancy compared with anemia in nonpregnant CKD.

Similar to anemia in healthy pregnancy, anemia in pregnant women with CKD will partly be explained by hemodilution and partly by the absolute iron deficiency caused by the increasing iron requirements during gestation.^32^^,^^41^, ^42^, ^43^ Furthermore, women with CKD are commonly prescribed aspirin in pregnancy for reducing the risk of preeclampsia.^100^^,^^101^ However, the potential impact of aspirin on gastrointestinal blood loss and subsequently worsening of iron deficiency has not been explored in this patient setting. In addition, the functional iron deficiency associated with CKD could contribute to the anemia in pregnant women with CKD.

Of the 3 important regulators of erythropoietic activity and iron homeostasis, it has been shown that pregnant women with CKD are not able to appropriately increase EPO levels, which in healthy pregnancy doubles between the first trimester and the end of the third trimester.^35^^,^^77^^,^^91^^,^^102^

For example, a case series of 5 pregnant women with CKD demonstrated an absence of EPO response to anemia.^103^ Until now, ERFE levels have not been investigated in pregnant women with CKD, but most likely will be decreased because ERFE production is stimulated by EPO. Finally, regarding hepcidin, it can be hypothesized that hepcidin may be relatively suppressed in pregnant women with CKD similarly to healthy pregnant women, but also that the inflammation in CKD may prevent adequate suppression of hepcidin, and thereby leads to disturbed iron uptake and mobilization.^51^ The latter is supported by several animal and human studies. For example, when inflammation was mimicked by administration of a hepcidin agonist in an animal study with pregnant mice,^51^ high doses of hepcidin agonist caused severe hypoferremia and anemia in both the mothers and embryo; whereas low doses of hepcidin agonist did not cause maternal anemia, but still adversely affected the embryo, causing anemia and tissue iron deficiency. Interestingly, increased levels of maternal hepcidin also caused adverse effects on embryo outcomes, including decreased placenta weight and birthweight, as seen in pregnant women with higher CKD stages.^10^^,^^102^^,^^104^ In a human cohort of pregnant women affected by inflammation and nutrient deficiencies, increased hepcidin levels were associated with a greater odds ratio of intrauterine growth restriction.^105^ In another human cohort with obesity as the inflammatory state, hepcidin levels were mildly increased in obese pregnant women compared with healthy pregnant women, but conflicting evidence was found on hematologic or iron variables in the mother or neonate.^106^, ^107^, ^108^ All together, these findings underscore the necessity to evaluate the effect of CKD on the course of hepcidin levels during pregnancy, maternal iron restriction and also on the offspring outcomes (Table 5).

**Table 5:** Research priorities for the management of anemia in pregnant women with CKD

Etiology and diagnosis of iron deficiency and anemia in pregnant women with CKD
1. Describe the variability in Hb and iron parameters in pregnant women with CKD by levels of eGFR, kidney transplantation, and kidney disease to increase understanding of the range of iron and Hb levels.
2. Increase knowledge on pathophysiology by conducting studies investigating Hb, iron parameters, sTFR, EPO, hepcidin, ERFE and IL-6 levels in pregnant women with and without CKD, and associate these to maternal and fetal outcomes.
3. Investigate cutoff levels for and changes in iron parameters and Hb during pregnancy associated with maternal and fetal outcomes and necessitating treatment.
Treatment of iron deficiency and anemia in pregnant women with CKD
4. Investigate the effectiveness of different dosing schedules with oral and i.v. iron formulations in all stages of CKD.
5. Collect international data regarding the safety and effectiveness of rhEPO in pregnancy.
6. Conduct a longitudinal cohort study of women with a range of CKD severity and pathology including postpartum relating RBC parameters, ERFE and sTfR to treatment with iron and/or rhEPO to maternal and fetal outcomes including anemia at birth in the offspring.
7. Conduct a trial using stable iron isotopes to gain insight in placental transfer of iron in pregnant women with CKD.
8. Investigate how maternal iron treatments impact the iron stores of the offspring at birth and the relation with bone metabolism.

CKD, chronic kidney disease; EPO, erythropoietin; ERFE, erythroferrone; Hb, hemoglobin; IL-6, interleukin-6; RBC, red blood cell; rhEPO, recombinant human erythropoietin; sTfR, soluble transferrin receptors.

#### Nutritional Deficiencies Related to Iron Deficiency and Anemia in Pregnant Women With CKD

Considering that macronutrient and micronutrient requirements are increased during pregnancy, pregnant women are at a high risk of developing (multiple) micronutrient deficiencies such as deficiencies in iron, folic acid (vitamin B9), and vitamin B12, among others; also partly because of suboptimal diet quality, and sometimes even despite use of supplements.^105^^,^^109^ However, adaptation of diet can help target certain deficiencies.^109^ For example, folic acid intake can be increased from a minimum of 4 weeks before conception in supplements but also by dietary fortification, also to reduce the risks for neural tube defects.^110^ As in CKD in general, also in pregnant women with CKD, interest has shifted from macronutrient intake to micronutrient intake and the combination of micronutrient intake by types of diet (e.g., Mediterranean diet, whole foods plant-based diet).^111^, ^112^, ^113^, ^114^, ^115^ In particular, pregnancy is a well-known physiological cause of hyperfiltration in which CKD is associated with an increase in proteinuria, but this increase could be diminished by diet.^112^^,^^116^ The Italian Study Group on Kidney and Pregnancy has performed the largest study on a plant-based, moderately protein-restricted diet in pregnant women with CKD to counterbalance this proteinuria.^112^ These 52 pregnant women with CKD receiving an intervention diet were compared with a propensity-score-matched cohort of CKD pregnancies on unrestricted diets. The goal was to control protein intake to 0.8 g/kg/d, with over 80% coming from plant-derived sources. Women in the control group had a significant increase in proteinuria during pregnancy, while the intervention group did not.^112^ However, when not planned safely, risks of a plant-based diet include pregnancy complications such as low birthweight and anemia caused by deficiencies in vitamin B12 and/or iron, because these micronutrients are already often deficient in pregnancy.^117^, ^118^, ^119^, ^120^ Some, therefore, do not recommend a low-protein diet.^33^ Especially in the case of iron deficiency, an additional risk could be increased uptake of heavy (toxic) metals in the intestine due to up-regulation of iron transporters that can take up (toxic) metals, which may also be consumed through contaminated vegetables and can have negative effects on the fetus by intrauterine growth restriction, preterm birth, and thyroid function.^115^^,^^121^, ^122^, ^123^

#### Treatment of Anemia and Iron Deficiency in Pregnant Women With CKD and Research Opportunities

At present, there is a paucity on clinical studies regarding anemia and iron deficiency in pregnant women with CKD. The lack of clinical studies is partly because of the relatively low frequency of pregnancy in women with CKD, particularly in the advanced stages. The heterogeneity of underlying diseases may further complicate the design of clinical studies or analysis of data. In Table 5, we provide research priorities following from the knowledge gaps outlined above and in this paragraph. However, to guide the clinical problem of anemia and iron deficiency in pregnant women with CKD, several guidelines, best practices, and reviews have formulated treatment approaches (Table 6).^33^^,^^61^, ^62^, ^63^, ^64^^,^^90^^,^^91^^,^^102^^,^^124^, ^125^, ^126^ These treatment approaches are summarized in a proposed flow-chart (Figure 3).

**Table 6:** Suggested treatment approaches from reviews and guidelines on anemia and iron deficiency in pregnant women with CKD

	Iron	Vitamin B9/B12	rhEPO	Blood transfusion
On CKD all stages				
Review 2016^102^	Oral iron considered safe	x	rhEPO considered safe	x
	I.V. iron likely to be safe		Required dose typically increases	
	Required dose typically increases			
Review 2018^33^	Oral iron is inexpensive & accessible	Consider B12 deficiency in women on low-protein diet	Supplementation with synthetic EPO may be required, even in the context of mild renal impairment	x
	Parenteral iron is generally considered safe in pregnancy & breastfeeding	For assessment of B12 deficiency use reference range for pregnancy	Women who required ESA before pregnancy, increased dose should be anticipated	
UK guideline 2019^62^	Oral iron: cheap and accessible	x	Recommend ESAs if indicated	x
	Recommend parenteral iron if indicated		Women who required ESA before pregnancy, increased dose should be anticipated	
Dutch guideline 2022^61^	Do not prescribe iron when Fe >500 μg/l and/or TSAT >30%	Exclude B9/B12 deficiencies	Consider rhEPO when Hb <10 g/dl	Consider leukocyte- depleted, Parvovirus B19-free, and cEK-compatible erythrocyte transfusions.
	Start with oral iron supplements		Consider increasing rhEPO when the woman already used rhEPO before pregnancy by 50%–100%. For most patients, a maximum dose of twice the normal starting dose is safe.	Perioperative transfusions if Hb parenteral iron if <7.3 g/dl, and consider if Hb <8 g/dl cannot be achieved in a stable situation. Consider if Hb <9.7 g/dl and major blood loss is expected.
	Consider starting iron with mild ID			
	In second/third trimester, switch to target levels with oral agents.			
	In case of i.v. iron: prescribe modern, stable iron supplements.			Consider transfusions in women on dialysis if Hb <9.7 g/dl and not expected to correct with iron/rhEPO.
				Consider the risk of allosensitization.
German guideline^64^	Treat with oral iron up to Hb <10.5 g/dl or with i.v. iron (Fe (III) derivates) up to Hb <8.5 g/dl			
Review 1994^90^	Treat with i.v. iron as needed by evaluating iron, total iron binding capacity and ferritin	Give 1 mg folate daily over and above usual supplement	Increase rhEPO dose by 50% as soon as pregnancy is recognized	x
Review 2007^91^	Treat with i.v. iron to keep iron saturation >30%	Give 1 mg/d folate	x	x
	Administer i.v. iron in small doses			
Comparative analysis 2014^124^	Increase dose of i.v. iron; typically administered weekly to maintain	x	Increase ESAs; typically administered weekly to maintain target Hb 10–11 g/dl	x
Best practices 2015^63^	Oral iron administration is safe in pregnant women on dialysis, whereas i.v. iron should be managed with care in dialysis mothers.	Folate and B12 supplementation should be tailored according to blood levels.	rhEPO is safe in pregnancy.	x
			ESA doses should be increased 50%–100% in attempt to achieve target Hb >10–11 g/dl with Ht >30%–35%.	
Review 2020^125^	Iron sucrose is considered safe in pregnancy and lactation	x	ESAs considered safe in pregnancy and lactation	x
	Increases in iron sucrose supplementation may be required	May need twice the typical dose to meet goal Hb 10–11 mg/dl		

CKD, chronic kidney disease; DD, dialysis-dependent; ESA, erythropoietin stimulating agent; Fe, ferritin; Hb, hemoglobin; ID, iron deficiency; rhEPO, recombinant human erythropoietin; TSAT, transferrin saturation; UK, United Kingdom.

**Figure 3:**
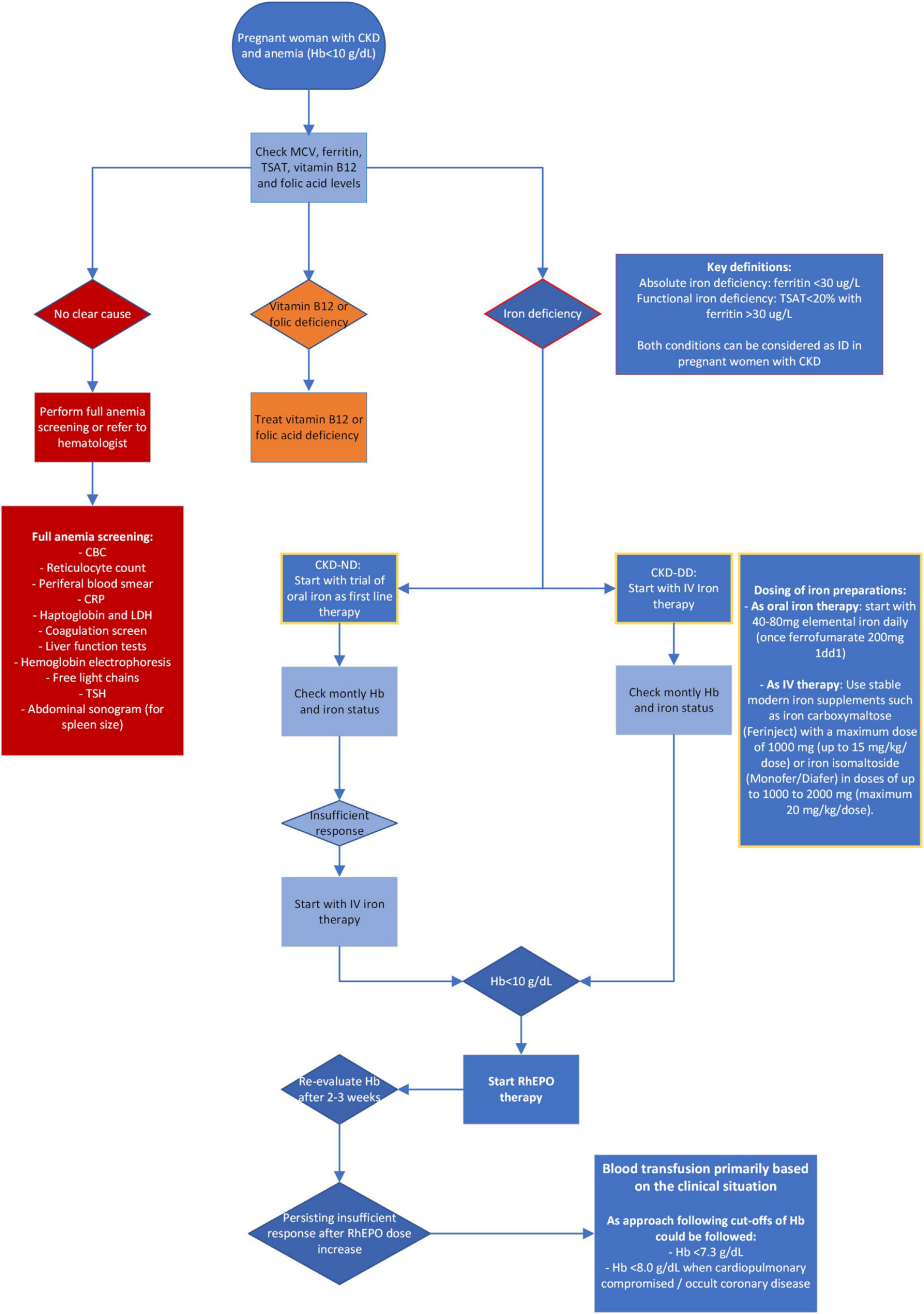
Proposed flow-chart for work-up and treatment of anemia during Pregnancy in CKD. CKD, chronic kidney disease.

In case of a low Hb, it is suggested to also check and supplement folic acid and vitamin B12 in CKD in general as well as in dialysis.^61^^,^^63^^,^^90^ Because a ferritin level <30 μg/l implies absolute iron deficiency also with concomitant CKD, and TSAT <20% has been validated as a good marker for functional iron deficiency in patients with chronic diseases,^3^^,^^127^ it seems reasonable to assume that pregnant women with CKD and anemia should be treated with iron when the ferritin level is <30 μg/l or when TSAT level is <20%, or even with mild iron deficiency, because of the increased iron need during pregnancy.^61^^,^^63^ No clear evidence exists regarding the optimum maintenance ferritin and TSAT levels with iron therapy, also not within the pregnancy field or the anemia in CKD field. In the latter, only the PIVOTAL study clearly showed that ferritin <700 μg/l or TSAT <40% can be safely used as upper limits for iron therapy. However, the optimal treatment targets of ferritin and TSAT in terms of safety, erythropoiesis stimulating agent dose reduction, and patient outcomes will need to be delineated in detail in the future, also for pregnant women with CKD. In case of iron deficiency, the guidelines and best practices recommend to start iron therapy with oral formulations because these are safe, cheap, and easily available.^61^, ^62^, ^63^, ^64^ When target levels are not reached with oral formulations, it is recommended to start i.v. iron formulations but only in the second and third trimester, although there is no evidence of teratogenicity or fetotoxicity if i.v. iron is given in the first trimester.^33^^,^^61^, ^62^, ^63^, ^64^^,^^102^ Frequent checks of Hb and iron parameters are recommended.^91^

Regarding treatment with EPO, animal studies with monkeys and sheep in late gestation and in *ex vivo* human placenta perfusion experiments showed that rhEPO did not pass the placenta.^128^^,^^129^ Scarce evidence from human case series and retrospective audits have not shown effects on teratogenicity in any trimesters of pregnancy; therefore, all guidelines and reports recommend that rhEPO can be safely given during the third and probably also first 2 trimesters of pregnancy.^33^^,^^34^^,^^48^^,^^77^^,^^90^^,^^91^^,^^102^^,^^129^, ^130^, ^131^, ^132^, ^133^ However, treatment with rhEPO may lead to (worsening of) maternal hypertension because of indirect vasoconstrictive effects.^134^ Incidence of hypertension and preeclampsia caused by rhEPO is reported to be low; however, the risk might be increased when blood viscosity or hematocrit increases; e.g., >5% in 4 weeks or becoming higher than 30% to 35%, as extrapolated from hemodialysis patients treated with rhEPO.^48^^,^^135^ Furthermore, all guidelines and reports recommend increasing the rhEPO dose between 50% to maximum 100% compared with preconception, because women with more advanced stages of CKD may have insufficient capacity for the gestational increase in EPO levels,^6^^,^^33^^,^^63^^,^^77^^,^^91^^,^^102^ and that rhEPO should be titrated every 2 to 3 weeks by Hb.^48^^,^^92^

Finally, regarding the role of blood transfusion in pregnant women with CKD, the goal in all potential candidates for future (kidney) transplantation and all kidney transplant recipients is to avoid blood transfusions because of the risk of allosensitization.^136^ Guidelines recommend delaying blood transfusion perioperatively until Hb <7.3 g/dl, or until 8.0 g/dl if the pregnant woman is cardiopulmonary compromised or has possible occult coronary disease.^61^

### Future Perspectives

Many questions on the mechanisms and optimal management of anemia and iron deficiency have not been answered for pregnant women with CKD. Pregnancy in women with CKD is becoming more frequent.^33^^,^^126^ Consequently, unraveling the pathophysiology of anemia in pregnant women with CKD is necessary, and could lead to improved treatment opportunities in these women and enhanced maternal and neonatal outcomes; therefore, we recommend prioritizing research in this topic.

## Disclosure

EN is a shareholder in Intrinsic LifeSciences, Silarus Pharma, and Disc Medicine; and has received consulting fees from Disc Medicine, FibroGen, AstraZeneca, GSK, Ionis Pharmaceuticals, Novo Nordisk, Protagonist, Vifor, Chiesi, RallyBio, and Shield Therapeutics.

KB has received consultant fees from AstraZeneca, GSK, Vertex, and Bayer.

MFE has declared receiving consultant fees from Vifor Pharma and Cablon Medical; serving on the Advisory Board for Cablon Medical and GlaxoSmithKline; and receiving speaker fees from Vifor Pharma, Pharmacosmos, and Astellas (all to employer). All the authors declared no competing interests.

## Author Contributions

MFCdJ conceptualized the study; MFCdJ, EN, PR, KB, and MFE wrote the original draft; and MFCdJ and MFE reviewed and edited the manuscript.
